# Complex cardiac implantable electronic device infections in Alberta, Canada: An epidemiologic cohort study of validated administrative data

**DOI:** 10.1017/ice.2023.48

**Published:** 2023-10

**Authors:** Teagan L. King, Derek S. Chew, Jenine Leal, Kristine Cannon, Derek V. Exner, Stephanie Smith, Oscar Larios, Kathryn Bush, Brian Yuen, Zuying Zhang, Elissa Rennert-May

**Affiliations:** 1 Department of Medicine, University of Calgary, Calgary, Alberta, Canada; 2 Department of Microbiology, Immunology, and Infectious Diseases, University of Calgary, Calgary, Alberta, Canada; 3 Department of Community Health Sciences, University of Cagary, Calgary, Alberta, Canada; 4 O’Brien Institute for Public Health, University of Calgary, Calgary, Alberta, Canada; 5 Snyder Institute for Chronic Diseases, University of Calgary, Calgary, Alberta, Canada; 6 Infection Prevention and Control, Alberta Health Services, Calgary, Alberta, Canada; 7 Libin Cardiovascular Institute, University of Calgary, Calgary, Alberta, Canada; 8 Department of Cardiac Sciences, University of Calgary, Calgary, Alberta, Canada; 9 Department of Medicine, University of Alberta, Edmonton, Alberta, Canada; 10 Department of Pathology and Laboratory Medicine, University of Calgary, Calgary, Alberta, Canada

## Abstract

**Objective:**

To establish the epidemiology of cardiac implantable electronic device (CIED) infections in Alberta, Canada, using validated administrative data.

**Design:**

Retrospective, population-based cohort study.

**Setting:**

Alberta Health Services is a province-wide health system that services all of Alberta, Canada.

**Participants:**

Adult patients who underwent first-time CIED implantation or generator replacement in Alberta, Canada, between January 1, 2011, and December 31, 2019.

**Methods:**

CIED implant patients were identified from the Paceart database. Patients who developed an infection within 1 year of the index procedure were identified through validated administrative data (International Classification of Diseases, Tenth Revision in Canada). Demographic characteristics of patients were summarized. Logistic regression models were used to analyze device type, comorbidities, and demographics associated with infection rates and mortality.

**Results:**

Among 27,830 CIED implants, there were 205 infections (0.74%). Having 2 or more comorbidities was associated with higher infection risk. Generator replacement procedures (odds ratio [OR], 0.55; 95% confidence interval [CI], 0.34–0.84; P = .008), age increase of every 10 years (OR, 0.73; 95% CI, 0.66–0.82; P ≤ .001), and index procedure after 2014 were associated with decreased risk. Comparing the infected to uninfected groups, the hospitalization rates were 2.63 compared to 0.69, and the mortality rates were 10.73% compared to 3.49%, respectively (P < .001).

**Conclusions:**

There is a slightly lower overall rate of CIED infections Alberta, Canada compared to previously described epidemiology. Implants after 2014, and generator replacements showed a decreased burden of infection. Patients with younger age, and 2 or more comorbidities are at greatest risk of CIED infection. The burden of hospitalization and mortality is substantially higher in infected patients.

Cardiac implantable electronic devices (CIEDs) include pacemakers (PMs), cardiac resynchronization therapy (CRT), and implantable cardiac defibrillators (ICDs). Infection is one of the most serious complications following CIED implantation. These infections include superficial pocket infections or vascular infections,^1,2^ and they are associated with substantial morbidity and mortality that typically require hospitalization and device removal.^1–7^ Mortality is reported as high as 17.2% with device-related infection.^4^ Additionally, CIED infections pose substantial economic burden to healthcare systems, increasing hospital length of stay and direct costs.^3^ The growing prevalence of CIED infections is concerning in the context of expanding CIED indications coupled with an aging, multimorbid population.^6,8–10^

At the hospital level, device-related infections have traditionally been determined through labor-intensive infection prevention and control (IPC) formal surveillance, which is considered the “gold standard.” This type of surveillance may be unachievable where resources are limited, such as smaller centers or rural areas without comprehensive IPC programs. An alternate method of infection surveillance is enabled by administrative data, which facilitates identification of CIED infection within health systems that do not routinely collect formal IPC surveillance data.^11^ The use of administrative data codes for identification of complex CIED infection was recently validated with 91% sensitivity and 99% specificity.^11^ These administrative codes have not previously been applied to assess the epidemiology of CIED infection following validation, particularly in the Canadian setting.

In this study, we sought to describe the epidemiology of complex infections following CIED implantation among patients in Alberta, Canada, by identifying CIED infections through validated administrative data. Our secondary objectives were to determine the risk factors for CIED infection and to assess the rates of rehospitalization and mortality in this contemporary patient cohort.

## Methods

### Study design

We conducted a population-based, retrospective cohort study of adult patients (ie, age >18 years) who underwent first-time CIED implantation or generator replacement between January 1, 2011, and December 31, 2019, in Alberta, Canada, which is a province of 4.2 million people served by a single healthcare system.

### Study population

Patients were included if they received a CIED during the study period and were followed in Alberta, Canada. Patients were followed for 1 year from their index implant procedure to identify CIED infections. We excluded patients under the age of 18, and if we were unable to identify key demographic information such as location, sex, or age.

### Data sources

#### Paceart

Adults who received a CIED during the study period were identified through the Paceart database, a province-wide repository of all CIED-related clinical encounters within Alberta, Canada, containing information on indications for device implantation, type of device, date of procedure, and basic patient demographics. Patients were included who received CIED implantations of all device types: PMs, ICDs, and CRT devices, including both first-time implants and generator replacements. We censored procedures repeated within 2 years of index surgical date to avoid double counting of encounters related to the same infection.

#### Alberta Health Services (AHS) analytics

AHS is a province-wide single health system servicing the province of Alberta, Canada. All residents of Alberta are eligible for public health insurance, and >99% participate. Each resident is assigned a personal health number that acts as a unique lifetime identifier enabling linkages to administrative health data.^12^ The AHS Enterprise Data Warehouse (EDW), accessed through AHS Analytics, contains the Discharge Abstract Database (DAD), National Ambulatory Care Reporting System (NACRS), Vital Statistics database, and Alberta Health Registry, for Alberta residents with an Alberta Health Care insurance plan. Hospitalizations were identified in the DAD, mortality data were collected from the Vital Statistics database, and comorbidity data were derived from both the DAD and the NACRS.

### Patient characteristics

We recorded patient characteristics including age, sex, device type, generator replacement, number of comorbidities, Elixhauser index, Pampalon deprivation index, urban or rural residence, and year of index procedure. The Elixhauser index is a set of comorbidity measures developed from administrative inpatient data to predict hospital costs, length of stay, and mortality; it is comprehensive compared to other comorbidities indices.^13^ The Pampalon deprivation index is a composite index that uses Canadian census data to create a measure of socioeconomic disparity.^14^ The Pampalon indicators are described in the footnote of Table 1. Urban versus rural residence data were determined based on address (ie, postal code).

**Table 1.** Baseline Patient Characteristics of Device Implantation and Infection

Variables	No. of Index Procedures	No. of Infection	Rate of Infection, %	*P* Value
**All**	27,830	205	0.737	
**Age**				.003
18–29 y	398	8	2.01	
30–39 y	541	8	1.48	
40–49 y	972	11	1.13	
50–59 y	2,665	21	0.788	
60–69 y	5,592	47	0.840	
70–79 y	8,036	55	0.684	
≥80 y	9,626	55	0.571	
**Sex**				.045
Male	16,865	144	0.854	
Female	9,791	61	0.623	
**Device type**				.001
CRT	3,361	43	1.28	
ICD	4,700	37	0.79	
LPM	75	0	0	
PM	19,598	125	0.64	
S-ICD	83	0	0	
**Generator replacement**				.001
No	20,781	175	0.842	
Yes	7,049	30	0.426	
**Pampalon deprivation index, material^a^**				.574
1 - Least deprived	4,278	26	0.608	
2	4,082	30	0.735	
3	4,499	30	0.667	
4	5,197	45	0.866	
5 – Most deprived	5,357	44	0.821	
**Pampalon deprivation index, social^b^**				.6776
1 – Least deprived	3,590	28	0.780	
2	3,107	20	0.644	
3	4,540	41	0.903	
4	5,680	39	0.687	
5 – Most deprived	6,496	47	0.724	
**Urban and rural residence**				.474
Urban	21,369	159	0.744	
Rural	4,414	38	0.861	
**Index procedure year**				<.001
2011	2,688	32	1.19	
2012	2,891	37	1.28	
2013	2,909	33	1.13	
2014	3,038	29	0.955	
2015	3,151	8	0.254	
2016	3,297	19	0.576	
2017	3,304	9	0.272	
2018	3,241	22	0.679	
2019	3,311	16	0.483	
**Comorbidities, no.**				<.001
0–1	5,973	7	0.117	
2–3	9,608	47	0.489	
4–5	6,530	48	0.735	
6+	5,719	103	1.80	
**Elixhauser index**				<.001
−10–0	1,350	2	0.148	
1–10	14,292	59	0.413	
11–20	9,223	79	0.857	
21–30	2,402	50	2.08	
31–40	492	10	2.03	
41–50	66	4	6.06	
51–58	5	1	20	

Note. PM, pacemaker; CRT, cardiac resynchronization therapy; ICD, implanted cardiac defibrillator; LPM, leadless pacemaker; S-ICD, subcutaneous implanted cardiac defibrillator

a Pampalon deprivation index material indicators: proportion of people aged 15 years and older with no high school diploma; population employment ratio of people aged 15 years and older; average income of people aged ≥15 years.

b Pampalon deprivation index social indicators: proportion of individuals aged 15 years and older living alone; proportion of individuals aged ≥15 years and whose marital status is separated, divorced or widowed; proportion of single-parent families.

### Outcomes

The primary outcome of interest was development of a complex CIED infection within 1 year of index procedure. Secondary outcomes were all-cause hospitalization and all-cause mortality at 1 year.

We linked Paceart data to the AHS DAD hospital admissions data and *International Classification of Diseases, Tenth Revision in Canada* (ICD-10-CA) codes to determine which patients developed a complex SSI within 1 year of the index procedure. This validated administrative algorithm, which demonstrated 91% sensitivity and 99% specificity, searched by ICD-10-CA codes^11^ for infection of an implantable cardiovascular or other device (T827, T857), infective endocarditis (I330, I339, I38, I398), and cellulitis of the chest wall or other unspecified site (L0330, L0339, L038, L039). The validated algorithm was based on definitions of complex CIED infections according to the Centre for Disease Control National Healthcare Safety Network (CDC/NHSN) protocols for surgical site infection (SSI), where ‘complex’ SSI included both deep incisional SSI of the fascia or muscle (excluding superficial SSI of skin), and organ-space SSI.^15^

### Statistical analyses

We summarized baseline patient characteristics as well as characteristics of infected versus uninfected patients, using a χ^2^ test of proportions. The number of CIED infections was divided by the total number of implants over the study period to obtain the CIED infection rate.

Comparative analysis of the infected versus uninfected cohorts were performed by constructing both univariable and multivariable logistic regression models for the outcome of infection at 1 year. Covariates were selected *a priori*, and included age, sex, device type, generator replacement, index year, and number of comorbidities. The fit of the model was assessed using the Akaike information criterion. Analyses were conducted using R version 4.1.0 statistical software (R Foundation for Statistical Computing, Vienna, Austria). Odds ratios and 95% confidence intervals were reported.

Ethics approval for this study was obtained from the University of Calgary Health Research Ethics Board (no. REB20-2186).

## Results

### Baseline characteristics

In total, 27,830 CIED implants were placed between 2011 and 2019 in Alberta, Canada, and 16,865 of these placed were in men (60.6%). The baseline characteristics of the cohort are summarized in Table 1. Of all CIED implants, 83.6% were in those aged ≥60 years. Pacemakers were the most common device type, comprising 70.4% of all insertions. Of all procedures, 25.3% were generator replacements. Patients were skewed toward higher degrees of social and material deprivation per the Pampalon index, and 76.8% of all patients lived in an urban setting. The Elixhauser comorbidity index ranged from −10 to 58, with a mean of 10.50 (SD, 7.50). The most prevalent comorbidities were hypertension (58.1%), diabetes mellitus (43.9%), and heart failure (39.4%). Pulmonary disease and renal failure were present in 16.9% and 9.8% of the population, respectively.

### Clinical outcomes

During the study period, 205 complex infections were identified at 1 year following device implantation, with an overall infection rate of 0.74%. The rate of infection did vary by age group, with the highest rate (2.01%) seen in those aged 18–29 years. The rate of infection decreased by each increasing increment of 10 years. Rates of infection also varied by device type: CRT devices (CRT-defibrillator and CRT-pacemaker types combined) at a rate of 1.28%, ICD at a rate of 0.79%, and pacemakers at a rate of 0.64% (*P* < .0001).

In univariable analysis, factors that were significant for increased odds of infection were male sex (OR, 1.37; 95% CI, 1.02–1.87; *P* = .038), CRT device type (OR, 2.02; 95% CI, 1.41–2.84; *P ≤* .001), 2 or more comorbidities, and Elixhauser index ≥11.

Factors that were associated with decreased odds of infection, in univariable analysis, included age increments of 10 years (OR, 0.83; 95% CI, 0.77–0.91; *P ≤* .001), a generator replacement procedure (OR, 0.5; 95% CI, 0.33–0.73; *P ≤* .001), and the procedure year of device implantation. Factors that were not associated with infection included material or social deprivation and urban or rural residence. Univariable analysis is summarized in Table 2.

**Table 2.** Univariable and Multivariable Odds Ratio of Infection

Factors	Unadjusted Logistic Model		Adjusted Logistic Model	
	Odds Ratio (95% CI)	*P* Value	Odds Ratio (95% CI)	*P* Value
Age, per 10 y	0.83 (0.77–0.91)	<.001	0.73 (0.66–0.82)	<.001
**Sex**				
Female	1	…	1	…
Male	1.37 (1.02–1.87)	.038	1.2 (0.86–1.68)	.292
**Device type**				
PM	1	…	1	…
CRT	2.02 (1.41–2.84)	<.001	1.44 (0.95–2.12)	.075
ICD	1.24 (0.84–1.77)	.259	0.86 (0.56–1.3)	.481
LPM	0 (0–48.73)	.978	…	…
S-ICD	0 (0–12.91)	.977	…	…
**Generator replacement**				
No	1	…	…	…
Yes	0.5 (0.33–0.73)	…	…	.008
**Pampalon deprivation index, material**				
1 – Least deprived	1	…	…	…
2	1.21 (0.72–2.06)	.477	…	…
3	1.1 (0.65–1.87)	.728	…	…
4	1.43 (0.89–2.35)	.149	…	…
5 – Most deprived	1.35 (0.84–2.23)	.222	…	…
**Pampalon deprivation index, social**				
1 – Least deprived	1	…	…	…
2	0.82 (0.46–1.46)	.51	…	…
3	1.16 (0.72–1.9)	.548	…	…
4	0.88 (0.54–1.44)	.606	…	…
5 – Most deprived	0.93 (0.58–1.5)	.752	…	…
**Urban and rural residence**				
Rural	1	…	…	…
Urban	0.86 (0.61–1.25)	.417	…	…
**Index procedure year**				
2011	1	…	1	…
2012	1.08 (0.67–1.74)	.763	0.93 (0.56–1.55)	.779
2013	0.95 (0.58–1.56)	.845	0.83 (0.49–1.4)	.48
2014	0.8 (0.48–1.33)	.386	0.55 (0.3–0.97)	.041
2015	0.21 (0.09–0.44)	<.001	0.18 (0.07–0.38)	<.001
2016	0.48 (0.27–0.84)	.012	0.37 (0.19–0.68)	.002
2017	0.23 (0.1–0.46)	<.001	0.22 (0.1–0.45)	<.001
2018	0.57 (0.32–0.97)	.042	0.51 (0.29–0.9)	.022
2019	0.4 (0.22–0.72)	.003	0.39 (0.2–0.72)	.003
**Comorbidities, no.**				
0–1	1	…	1	…
2–3	4.19 (2.02–10.16)	<.001	4.16 (1.78–12.15)	.003
4–5	6.31 (3.05–15.3)	<.001	7.59 (3.26–22.17)	<.001
6+	15.63 (7.83–37.1)	<.001	18.15 (8.02–52.17)	<.001
**Elixhauser index**				
−10–0	1	…	…	…
1–10	2.79 (0.87–17.06)	.153	…	…
11–20	5.82 (1.83–35.42)	.014	…	…
21–30	14.33 (4.44–87.71)	<.001	…	…
31–40	13.98 (3.67–91.19)	.001	…	…
41–50	43.48 (8.33–318)	<.001	…	…
51–58	168.5 (7.03–2,173.94)	<.001	…	…

Note. CI, confidence interval; PM, pacemaker; CRT, cardiac resynchronization therapy; ICD, implanted cardiac defibrillator; LPM, leadless pacemaker; S-ICD, subcutaneous implanted cardiac defibrillator.

**Fig. 1.**
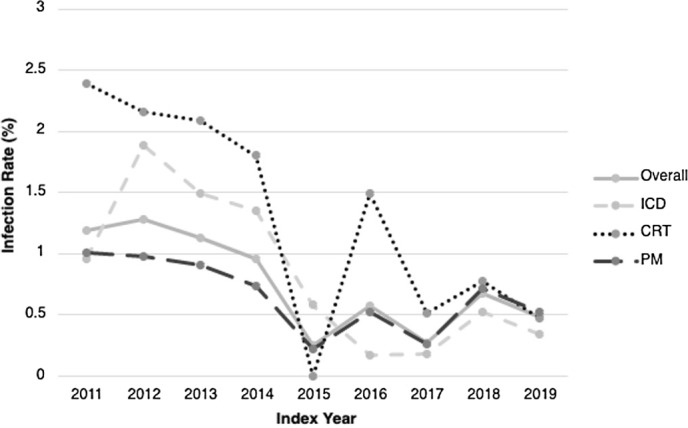
Infection rate (%) by index procedure year comparing device types. Trend of number of infections over total number of implants for each index procedure year, compared by individual device types and overall (all devices). Note. CRT, cardiac resynchronization therapy; PM, pacemaker; ICD, implantable cardiac defibrillator.

In multivariable analysis, the variable that remained significantly associated with CIED infections was having 2 or more comorbidities. Elixhauser index was no longer significant. Generator replacement procedures (OR, 0.55, 95% CI, 0.34–0.84; *P* = .008), age increments of 10 years (OR, 0.73; 95% CI, 0.66–0.82; *P ≤* .001), and index procedure after 2014 were associated with decreased risk of infection. Multivariable analysis is summarized in Table 2. Figure 1 shows the infection rates by index procedure year.

The most common comorbidities, including hypertension, heart failure, and complicated diabetes, were independently associated with increased infection risk, as were chronic pulmonary disease, and renal failure. The conditions associated with the greatest risk of infection, in descending order, were acquired immunodeficiency syndrome, valvular disease, substance use disorder, liver disease, fluid and electrolyte disorders, and complicated hypertension. Table 3 provides a summary of the common comorbidities and their associated risk, and complete comorbidity data are available in Supplementary Material Table S1 (online).

**Table 3.** Prevalence of Common Comorbidities and Relationship to Infection

Comorbidities	No. Patients	No. Infection	Rate Infection, %	Odds Ratio Infection (95% CI)	*P* Value
Hypertension, uncomplicated	15,849	136	0.858	1.37 (1.02–1.86)	.036
Congestive heart failure	10,953	125	1.14	2.37 (1.78–3.18)	<.001
Diabetes, complicated	6,646	66	0.993	1.5 (1.11–2.02)	.008
Diabetes, uncomplicated	5,585	48	0.859	1.2 (0.85–1.65)	.284
Chronic pulmonary disorders	4,705	63	1.34	2.19 (1.6–2.94)	<.001
Renal failure	2,740	47	1.72	2.7 (1.92–3.74)	<.001
Hypertension, complicated	317	8	2.52	3.62 (1.63–6.95)	<.001

Note. CI, confidence interval.

Patients who developed infection were more likely to require hospital admission compared to those who did not develop infection in the year following the index procedure. Of the 27,625 patients without infection, the mean number of admissions was 0.69, compared to 2.63 admissions among the 205 patients with infection. With respect to mortality, patients who developed a complex infection were more likely to die within the year following implantation than those who did not develop infection. Of the patients where mortality data were available, 22 of 175 patients with infection died, compared to 964 deaths in the 24,622 patients without infection. The death rate in the infected group was 10.73% compared to 3.49% in the uninfected group (*P* < .001).

## Discussion

Between 2011 and 2019, there were 27,830 total CIED implants in Alberta, Canada, with a rate of infection of 0.74%. This is a comprehensive, population-based epidemiological evaluation of all CIED infections in Alberta, enabled by validated administrative data. In a large systematic review of CIED infections up to 2013,^16^ an infection rate of 1.2% across retrospective studies was reported. A cohort in Ontario, Canada, reported an overall infection rate of 1.2%,^4^ and a similar study in the United States in 2016 found rates as high as 4.2% in administrative, retrospective data^3^; however, the administrative data in these studies were not validated. Our data show a slightly lower overall infection rate than previous studies.

Previous studies reported rates of CIED infection to be increasing out of proportion to device implantation, and over time.^6,8,9^ One study noted an infection rate that increased from 1.53% in 2004 to 2.41% in 2008 (*P* < .001).^8^ Our data showed a significant decrease in infection rates among the study population from 2014 forward. We offer several hypotheses for this finding. Firstly, there have been several recent clinical trials on prevention of CIED infection, namely PADIT^17^ and WRAP-IT.^18^ Sites within Alberta were enrolled in the PADIT trial between 2011 and 2014. Implementation of rigorous trial protocols and infection prevention measures, including an expanded protocol of preoperative cefazolin and vancomycin, postoperative cephalexin, and an intraprocedural wound-pocket wash with bacitracin, may have influenced a decreasing trend of infection within our cohort following trial completion. Secondly, formal surveillance of CIED infections increased in the Calgary region in recent years; the largest provincial zone, servicing ∼1.5 million people. Formal SSI surveillance was implemented in June 2012 and expanded over several years to include stakeholders outside the operating room during the study period, including the electrophysiology suite where many implants are performed. As part of this enhanced surveillance, interventions including surgical bundles and checklists (eg, prophylaxis timing) were introduced, contributing to decreased SSIs within the AHS. The SSI rate in 2011–2012 was 1.0 cases per 100 procedures compared to 0.49 in 2019–2020 (K. Cannon, personal communication, December 15, 2022). We may begin to see broader regression in infection trends as newer studies emerge.

Few other epidemiologic studies have examined markers of inequities, including urban versus rural residence, and socioeconomic disparity, as they relate to risk of infection. In our cohort, infectious outcomes did not differ based on urban or rural living, or Pampalon index, a marker of socioeconomic disparity.^14^ This finding is unique. Within a cohort in Ontario, residing in a higher income neighborhood was associated with greater infection risk, as was having a device implanted or replaced in an urban teaching hospital.^4^ Within a large US cohort in 2016, higher income quintiles had higher infection-related costs; and urban or rural location of care made no difference in infection risk or costs.^3^ One explanation for these findings is that patients of higher income are more likely to undergo device implantation despite higher overall risk profiles. Smaller, rural centers that perform fewer procedures might be expected to have higher infection rates; however, we can speculate that urban teaching hospitals have the greatest infectious complications due to greater numbers of operators in training,^2,16^ increased procedural time,^16^ and higher case complexity seen in urban teaching facilities. Our findings of relative equity across income quintiles and place of residence, of the patients that accessed care and when Pampalon index was available, may reflect the single health system model, including CIED clinics, that creates integrated service delivery in Alberta regardless of income or residence. Alternatively, more marginalized, and geographically isolated groups may simply not be represented due to incomplete census data, resulting in skewed findings. We advocate for future studies to include measures of disparity because this may be an underrecognized issue.

Increasing age was associated with reduced infection risk. This finding is aligned with several other reported findings, including the PADIT trial,^17^ in which lower age conferred higher risk in the validated prediction tool.^19^ Also, a large Danish study of 46,299 patients showed a clear reduction in risk with advancing age.^20^ The precise biologic explanation for this effect is not known. Researchers have postulated that this effect may be secondary to reduced immune responses in advanced age, less firm connective tissue, or lower likelihood of performing implants in higher-risk clinical scenarios.^20^

We noted a significantly increased risk of infection among men in univariable analysis. This finding has been reported in other studies,^3,16^ and needs to be interpreted cautiously because significant enrollment differences remain between men and women regarding device therapy.^21^ Women may have higher mortality once infected,^22^ and concern has been raised around sex-biased care, with findings of reduced length of stay and healthcare expenditures for women with device infections in one study.^3^ Analysis of a larger population would allow for more meaningful detection of differences between sexes. Further exploration of length of stay and health expenditures as they relate to sex would be valuable.

We identified several key findings regarding patient comorbidities and infection risk. Importantly, renal failure and congestive heart failure confer a 3-fold risk of infection over baseline, and complicated hypertension, valvular disease, substance use disorder, valvular and liver disease confer greater than a four-fold risk. These data can refine shared decision-making with patients regarding individualized procedural risk. These findings should also prompt operators to consider stricter IPC bundles for higher-risk patients, such as operating room practices, patient flow, and patient-specific measures such as antibiotic envelopes at the time of device insertion.^23^

In this study, patients who underwent a generator replacement had lower infection rates than first-time procedures (OR, 0.55; 95% CI, 0.34–0.84; *P* = .008). Other studies had similar findings in the meta-analysis by Polyzos,^16^ where 5 of 20 studies examining this procedure found that generator replacement was either protective or not associated with increased infection but was a risk factor in pooled analysis (OR, 1.74; 95% CI, 1.22–2.49).^16^ This may be due to shorter procedural time for generator change than first-time insertion, as well as the possibility that revision procedures stimulate higher level of attention to infection control measures by proceduralists.

Our study had several strengths. This was the largest epidemiologic review of CIED infections in Alberta, Canada, to date, and one of few Canadian studies that contributes to understanding these SSIs. Perhaps most importantly, our method of identifying infections using administrative data was recently validated through a comparative analysis of established ICD-10-CA algorithms and was proven to have comparable validity to the reference standard of IPC surveillance methods.^11^ This now-validated method of identifying CIED infections can act as a framework for future surveillance and research.

Our study also had several limitations. It was subject to limitations inherent in a population-based study using administrative data. Administrative data are subject to miscoding; however, the validated data were shown to be 91% sensitive and 99% specific, and they allowed us to characterize an entire population, which would have otherwise been prohibitive due to cost and difficulty in data collection. Another limitation of the study pertained to the retrospective nature of administrative data, and the inability to exclude possible unmeasured factors that may have influenced the relationship between infection, hospitalization, and mortality. We included only patient encounters accessible through discharge data and emergency department visits; thus, meaning superficial infections were excluded. However, these types of infections do not require comparable healthcare resources nor cause substantial patient morbidity. We were unable to determine the Pampalon index for nearly 8,000 patients in our cohort, likely because census data was incomplete. We chose to follow patients for 1 year from the index procedure so some late infections may have been missed; however, this time frame balances adequate surveillance while minimizing capturing infections unrelated to the initial implant.

In summary, we detailed the epidemiology of CIED infections from a population within Alberta, Canada. Our findings were aligned with much of the current body of literature, redemonstrating that patients with younger age and 2 or more comorbidities remain at greatest risk of CIED infection and that the burden of mortality is substantially higher with CIED infection. A novel finding was that our overall infection burden was slightly lower than previously described rates, and patients with implants after 2014 showed a decreasing trend in burden of infection, which may be related to enhanced IPC efforts in Alberta. Further analysis of the microbiology, costs, and implications of these infections at the system level is warranted.

## Supplementary material

For supplementary material accompanying this paper visit https://doi.org/10.1017/ice.2023.48. click here to view supplementary material
