# Supplementary material for: Complex cardiac implantable electronic device infections in Alberta, Canada: An epidemiologic cohort study of validated administrative data
Source: Infect Control Hosp Epidemiol. 2023 May 15;44(10):1607–13. doi: 10.1017/ice.2023.48 (PMC10587380; doi:10.1017/ice.2023.48)
Supplement: Supplementary file 1 [file S0899823X2300048Xsup001.docx]

**APPENDIX I**

**Supplemental Table S1:** Demographics of Comorbidities and Relationship to Infection

| **Comorbidities** | **No. Patients** | **No. Infection** | **Rate Infection (%)** | **Odds Ratio Infection (95% CI)** | **P-Value** |
| --- | --- | --- | --- | --- | --- |
| Cardiac Arrhythmias | 25657 | 199 | 0.776 | 2.86 (1.09-11.59) | 0.071 |
| Hypertension, uncomplicated | 15849 | 136 | 0.858 | 1.37 (1.02-1.86) | 0.036 |
| Congestive Heart Failure | 10953 | 125 | 1.14 | 2.37 (1.78-3.18) | <0.001 |
| Diabetes, complicated | 6646 | 66 | 0.993 | 1.5 (1.11-2.02) | 0.008 |
| Diabetes, uncomplicated | 5585 | 48 | 0.859 | 1.2 (0.85-1.65) | 0.284 |
| Fluid/Electrolyte Disorders | 4800 | 90 | 1.88 | 3.89 (2.93-5.16) | <0.001 |
| Chronic Pulmonary Disorders | 4705 | 63 | 1.34 | 2.19 (1.6-2.94) | <0.001 |
| Valvular Disease | 3835 | 100 | 2.61 | 6.07 (4.58-8.06) | <0.001 |
| Renal Failure | 2740 | 47 | 1.72 | 2.7 (1.92-3.74) | <0.001 |
| Peripheral Vascular Disorders | 1879 | 35 | 1.86 | 2.9 (1.98-4.14) | <0.001 |
| Hypothyroidism | 1835 | 20 | 1.09 | 1.53 (0.93-2.38) | 0.072 |
| Pulmonary Circulation Disorders | 1833 | 37 | 2.02 | 3.09 (2.11-4.39) | <0.001 |
| Solid tumor | 1742 | 16 | 0.918 | 1.28 (0.73-2.07) | 0.349 |
| Obesity | 1573 | 16 | 1.02 | 1.42 (0.81-2.29) | 0.184 |
| Other Neurological Disorders | 1549 | 21 | 1.36 | 1.96 (1.21-3.01) | 0.004 |
| Depression | 1420 | 19 | 1.34 | 1.93 (1.16-3.02) | 0.007 |
| Coagulopathy | 1333 | 26 | 1.95 | 2.92 (1.89-4.35) | <0.001 |
| Anemia, Deficiency | 1177 | 13 | 1.10 | 1.53 (0.83-2.59) | 0.14 |
| Weight Loss | 924 | 19 | 2.06 | 3.01 (1.81-4.72) | <0.001 |
| Alcohol | 685 | 8 | 1.17 | 1.62 (0.73-3.08) | 0.186 |
| Rheumatoid Arthritis | 619 | 11 | 1.78 | 2.52 (1.28-4.44) | 0.003 |
| Liver Disease | 611 | 21 | 3.44 | 5 (3.04-7.81) | <0.001 |
| Metastatic cancer | 522 | 8 | 1.53 | 2.14 (0.96-4.08) | 0.036 |
| Paralysis | 402 | 8 | 1.99 | 2.79 (1.25-5.34) | 0.005 |
| Peptic Ulcer Disease | 384 | 4 | 1.04 | 1.43 (0.44-3.38) | 0.484 |
| Hypertension, complicated | 317 | 8 | 2.52 | 3.62 (1.63-6.95) | <0.001 |
| Anemia, Blood Loss | 286 | 3 | 1.05 | 1.42 (0.35-3.77) | 0.545 |
| Substance Use Disorder | 267 | 11 | 4.12 | 6.07 (3.07-10.77) | <0.001 |
| Lymphoma | 225 | 3 | 1.33 | 1.85 (0.46-4.92) | 0.292 |
| Psychoses | 135 | 0 | 0 | 0 (0-0) | 0.971 |
| AIDS | 17 | 1 | 5.88 | 8.28 (0.46-40.87) | 0.041 |
